# One-Year Follow-Up after Multimodal Prehabilitation Interventions in Radical Cystectomy

**DOI:** 10.3390/cancers15245785

**Published:** 2023-12-10

**Authors:** Bente Thoft Jensen, Jørgen Bjerggaard Jensen

**Affiliations:** 1 Department of Urology, Aarhus University Hospital, 8200 Aarhus, Denmark; bjerggaard@skeby.rm.dk; 2 Department of Public Health, Aarhus University, 8200 Aarhus, Denmark; 3 Department of Clinical Medicine, Aarhus University, 8200 Aarhus, Denmark

**Keywords:** prehabilitation, radical cystectomy, bladder cancer, exercises, nutritional care, survivorship, ERAS

## Abstract

**Simple Summary:**

This study group has previously published and documented that multimodal prehabilitation (enhanced optimization of physical function and adjustment of modifiable medical issues) before major bladder surgery is feasible and leads to a positive change in patients’ fitness and functional status from a short-term perspective after surgery. However, long-term follow-ups after prehabilitation have not yet been published. Thus, this study aims to evaluate 1-year results on the efficacy of physical prehabilitation on functional capacity and nutritional recovery to inform the bladder cancer community of the potential of prehabilitation. The results show that prehabilitation in major bladder cancer surgery can significantly improve physical function and support the maintenance of nutritional status one year after major bladder cancer surgery. The results promote the gradual recognition that early restoration of physical function is vital to a full recovery.

**Abstract:**

Multimodal prehabilitation is the process of enhancing physiological, nutritional, and psychological resilience to increase patients’ functional capacity before major cancer surgery and aims to empower the patient to withstand the pending stress of major surgery and ultimately to improve long-term outcomes. The effect of physical prehabilitation to counteract the physical decline in surgical cancer patients has been documented; however, long-term results have not yet been published. This follow-up study aims to evaluate 1-year results on the efficacy of physical prehabilitation after bladder cancer surgery. The efficacy of prehabilitation was measured over the course of 1 year in 107 patients randomized to (1) pre- and rehabilitation or (2) standard care divided by *n* = 50 in the intervention (I) and *n* = 57 in the standard group (S). Physical function was measured by muscle leg power, and nutritional status was expressed with handgrip strength. Prehabilitation in major bladder cancer surgery can significantly improve physical function with 19.8 Watt/kg (*p* = 0.04), lean body mass (*p* = 0.047) and body cell mass (*p* = 0.03), and regained nutritional status one year after surgery. The results demonstrate that the restoration of physical function is vital to a full recovery.

## 1. Introduction

Radical cystectomy (RC) with pelvic lymphadenectomy remains the standard of care for treating muscle-invasive bladder cancer (MIBC) and high-grade non-muscle invasive disease (NMIBC) [1]. The surgical procedure is complex and associated with a significant postoperative morbidity with an all-course complication rate of up to 90% within the first 90 days post-surgery and mortality rates of up to 5% [2].

Despite the Enhanced Recovery after Surgery (ERAS) protocols, including the significant improvement in anesthetic protocols, minimal-invasive surgical techniques, and optimized postoperative care protocols, the rate of postoperative complications remains high. Therefore, prehabilitation has become an emergent clinical focus area with the specific aim to reduce modifiable risk factors before surgery and optimize functional status [3,4]. In an ERAS context, multimodal prehabilitation is the process of enhancing physiological, nutritional, and psychological resilience to increase patients’ functional capacity before major cancer surgery [5,6] and aims to empower the patient to withstand the pending stress of major surgery, improve the rate of postoperative recovery, and ultimately, through behavioral change, improve long-term health outcomes and survivorship [7,8].

The positive effect of physical prehabilitation to counteract the physical decline in health status in surgical cancer patients has been previously documented across different cancer diagnoses and stages but lacks long-term follow-ups across cancer sites [3,6]. The extended ERAS pathway, including physical prehabilitation, is not yet considered the gold standard, possibly due to concerns about delays in RC. However, leading urological societies and experts recognize the emerging evidence and the potential of prehabilitation [9,10].

This study group has previously published and documented in an RCT study that multimodal prehabilitation before RC was feasible and led to a positive and significant change in patients’ fitness and functional status compared with the standard protocols after surgery. Moreover, specific health-related quality-of-life items such as respiratory and abdominal function, as defined by the European Organization for Research and Treatment of Cancer (EORTC), were improved from a short-term perspective [3,11,12,13]. Currently, only a short-term follow-up of eight weeks has been reported after multimodal prehabilitation. Thus, this RCT study aimed to evaluate 1–year results on the efficacy of physical prehabilitation on functional capacity and nutritional recovery to further inform the bladder cancer community of the potential of prehabilitation to support long-term recovery and survivorship. 

### The Aim

To evaluate the long-term outcome of an RCT study testing multimodal prehabilitation intervention on physical and nutritional recovery after radical cystectomy.

## 2. Material and Methods

An RCT study conducted at Aarhus University Hospital (DK) investigated the efficacy of a pre- *and* postoperative multidisciplinary pre- and rehabilitation program following RC from 2011–2014, including one-year follow-up data for post hoc analysis (NCT01329107). The study included patients scheduled for RC because of localized MIBC or high-risk NMIBC during the study period, leaving 50 patients in the intervention group (nI = 50) and 57 patients in the standard group (ns = 57). Further information on the inclusion, randomization process, and Consort flowchart has been previously published [14] (Table 1).

### 2.1. The Prehabilitation Program (Intervention)

#### 2.1.1. The Physical Component

Specialized physiotherapists instructed the intervention group in a preoperative homebased program consisting of six general exercises to empower muscle strength and endurance. The exercise training program targeted the major muscle groups used for early mobilization and everyday activities such as mobilization, getting in and out of bed, chair raise performance, stair climbing, and gait. On average, it took 25 min to perform the exercises (10 to 15 repetitions each), which were recommended to be performed twice a day for two weeks before surgery. To meet the requirements of physical activity for a minimum of 30 min daily with moderate intensity as internationally recommended [15], a step-trainer was supplied to the intervention group, and the achievements were logged daily in a diary. Enhanced mobilization was defined by everyday goals concerning walking distance and hours out of bed.

Patients in the standard group did not receive prehabilitation intervention but only the standardized national recommendation regarding physical activity and nutritional care when in line with international recommendations [15,16]. Moreover, patients were postoperatively mobilized according to the standardized ERAS protocol.

#### 2.1.2. Nutritional Component

Because of the well-known effect of preoperative oral supplements, *all* patients received oral supplements in accordance with the European Society of Clinical Nutrition and Metabolism (ESBEN) guidelines before cancer surgery [17]. All patients underwent nutritional screening and were informed about individual sufficient protein intake ahead of surgery and the immediate need of early oral protein intake postoperatively as per ERAS RC protocol [18]. Individual nutrient requirements were calculated as a minimum of 1.2 g of protein per kilogram of body weight, as per the ESPEN guidelines for surgical patients [16]. All patients were given on-market oral nutritional supplements (Nutridrink Protein^®^, N. V. Nutricia) in addition to their normal diet. They were asked to drink two 200 mL bottles of this hypocaloric formula (1.5 kcal/mL) daily for two weeks before surgery. Each bottle contained 20 g of protein. As nutritional care was a part of the standard care program, all patients were able to collect a full bag of oral supplements covering the period before surgery. In addition, all patients were instructed to keep a food diary as well as report the number of oral supplements they could tolerate [14].

#### 2.1.3. Follow-Up Care

The same framework of exercises and enhanced mobilization were included in the postoperative care for patients in the intervention group and in the individual discharge plan [14]. While no guidelines or international recommendations existed for optimal follow-up care after RC [19], each patient was encouraged to perform self-care, continue the focus on choosing protein as the nutrient of interest, and continue the daily exercises at home, including the 30 min aerobic activities in line with general international recommendations for physical activity [15]. Thus, there were no scheduled follow-up instructions after discharge. In the case of further interest or need of empowerment, patients were recommended to join the facilities in the primary care setting or subscribe to training events performed by the national cancer society.

### 2.2. Measurements

All patients underwent a baseline assessment including physical function, nutritional status, and body composition (bioimpedance). All tests were repeated the day before surgery, at discharge, and five weeks, four months, and twelve months postoperatively. Leg extension power was chosen as a proxy for physical function because it correlates with the ability to perform physical activity [20]. Physical function was measured using Nottingham Leg Extensor Power Rig Software^®^ (2010.v1.0) and expressed in watts and normalized to body weight (watts per kilo).

Handgrip strength (isometric muscle strength) is associated with nutritional status and loss of functional status and is a common predictor of postoperative complications [21]. In both the above-mentioned measurements, a standardized test protocol was used that was previously reported [11].

Bioelectrical impedance was used to measure the change in body composition during the study period, including body mass index (BMI), fat mass in kilograms, and the percentage of total body weight and lean body mass [22]. Preoperatively, all patients self-reported oral intake using a personal nutritional diary, measuring kilojoules (KJ) and protein intake (grams) over the course of two weeks, including oral supplements. Postoperative oral intake as well as intravenous supplements were reported with an electronic medical record. Adherence to the program was 66% and has been previously reported [11].

### 2.3. Statistics

The efficacy of physical prehabilitation was expressed as the mean differences in mean muscle power, handgrip strength, and bioimpedance parameters between treatment groups at the time of surgery and at every oncological follow-up appointment for one year. Student’s *t*-test was used to test for statistical differences in muscle power, handgrip strength, and bioimpedance parameters between treatment groups. A *p*-value of < 0.05 was considered significant. All statistical analyses were performed using STATA 13 [23].

## 3. Results

**Figure 1:**
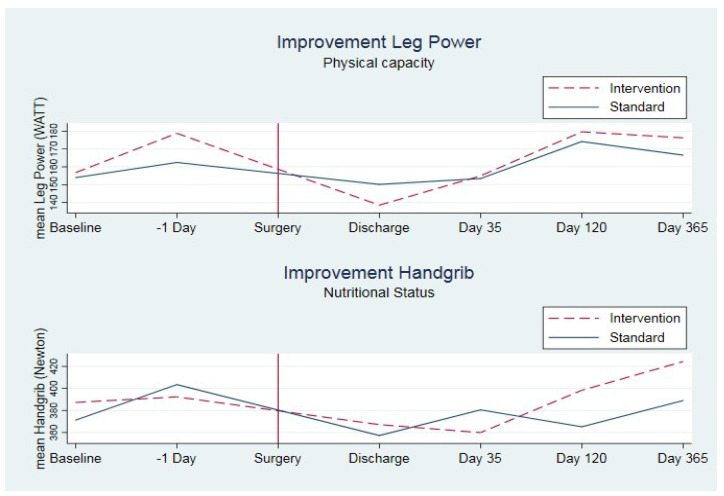
Functional status over the course of 1 year after radical cystectomy measured with leg muscle power and handgrip strength in 107 patients undergoing radical cystectomy at Aarhus University Hospital (2011–2014) (intention-to-treat approach).

There was a sustainable long-term effect of the prehabilitation interventions over the course of one year after RC (Figure 1). Compared to the standard group, the intervention group demonstrated a significant improvement in muscle power at the time of surgery (*p* = 0.001), at four months (*p* = 0.01), and at 12 months postoperatively (*p* = 0.04). However, the standard group presented higher muscle power at discharge (Table 2).

Handgrip strength was significantly higher in the standard group at surgery compared with the intervention group (*p* = 0.04) and five weeks postoperatively (*p* = 0.04). No significant difference between the groups was seen at four and twelve months postoperatively, although noteworthily, a higher improvement was observed in the intervention group at both four and twelve months (Table 2).

The bioimpedance parameter over the course of 12 months reflected the findings in handgrip strength and muscle power measurement. A significant improvement in lean body mass (*p* = 0.047) and total body cells (*p* = 0.03) was reported at 12 months compared with those of the standard group accompanied by a significant reduction in the fat mass of total body weight at discharge (*p* = 0.04) and at day 35 (*p* = 0.04) and maintained a lower value throughout the follow-up period (Table 3). No differences were found in the mean intake of energy or preoperative and postoperative protein (oral or intravenously) as previously reported [11].

## 4. Discussion

To our knowledge, this was the first randomized study reporting a 1-year follow-up on the efficacy of prehabilitation intervention in major bladder cancer surgery on physical functioning. Other studies in this specific population have reported follow-up outcomes after 8 weeks at maximum [3,12,24].

In this study, the intervention group demonstrated a significant positive improvement in muscle power at the time of surgery (*p* = 0.001) and at four months (*p* = 0.01) and 12 months postoperatively (*p* = 0.04) compared with the standard group (Table 2). In contrast, the standard group maintained a baseline level throughout the follow-up period. The results confirmed that prehabilitation, which is also called “the window of opportunity” to improve lifestyle, promotes early restoration of physical function and is sustainable up to one year after surgery [25].

Exercises provide the best anabolic stimulus, and nutrition potentiates the muscle protein response [16]. In this study, every patient underwent nutritional screening, and protein requirements were calculated as 1.2 g of protein per kilogram of body weight, as per ESPEN guidelines for surgical patients. However, this may have been beyond their actual needs as indicated in Figure 1, where the intervention group had a significantly lower handgrip strength (a proxy for nutritional status) after the end of the prehabilitation at the time of surgery (Table 2). Nutritional status was not restored in the intervention group opposed to the standard group before four months after surgery. A recent evaluation of dietary protein requirements in cancer surgery suggested that intakes in the range of at least 1.2 to 1.6 g/(kg day) were required to support optimal health in aging, frail patients like those in the RC population [26,27]. This could explain the unexpected loss of handgrip strength, suggesting that the intervention group lacked sufficient protein intake due to progressing physical activity compared with that in the standard group in both the pre- and rehabilitation period (Figure 1). The bioimpedance parameter over the course of 12 months reflected the findings in handgrip strength and muscle power measurements. A significant improvement in lean body mass (*p* = 0.047) and total body cells (*p* = 0.03) was reported at 12 months compared to the standard, accompanied by a significant reduction in the fat mass of total body weight at discharge (*p* = 0.04) and at day 35 (*p* = 0.04) and maintained a lower value throughout the follow-up period (Table 3). The same result was documented in another comprehensive cancer center with a high volume of RCs [28].

The same pattern was observed in other prehabilitation studies, e.g., in colorectal cancer surgery, where Gillies et al. argued that preoperative exercise alone was insufficient to improve surgical outcomes. Thus, it is important to recognize the synergistic effect of the sufficient nutrition- and exercise-induced stimulation of muscle protein synthesis that influences the protein balance to a greater extent compared to either nutrition or exercise alone [27].

The provision of adequate total protein intake must be the focus of nutritional pre- and rehabilitation interventions, especially when it is estimated that 62% of patients with advanced bladder cancer undergoing RC are frail, which is associated with a seven-fold increased risk of a severe complication or death 1 year after RC [29].

Conjointly, cancer-induced impairments and risk factors associated with the aging process, such as a high comorbidity index, polypharmacy, and cognitive and physical impairment, are associated with all-cause mortality and represent a further challenge in relation to recovery after RC [30]. Thus, prevention and health promotion interventions like prehabilitation are warranted, and these long-term results combined with accumulating evidence suggest that prehabilitation has a future role to prevent physical decline and further enhance the restoration of physical function after RC [3,31]. Only a few RCT studies have been performed in this field, and future prehabilitation studies may benefit from a standardized set of criteria to ensure transparency between the programs and settings. This could include patient selection, the specific content, and thorough adherence tracking as suggested by Briggs et al. [31]. Today, the prehabilitation interventions reported in the literature are heterogenic; thus, a consensus is required to determine which exercise program or outcomes should be measured and when to improve evidence and the knowledgebase. Finally, surgical societies should establish a consensus on how outcomes should be reported. In alignment with these recommendations, the outcome of exercise and nutrition interventions within prehabilitation programs should be more stringent and transparent for clinical efficacy and enhance implementation.

## 5. Limitations

This was the first RCT study to report a 1-year follow-up on the efficacy of a multimodal pre- and rehabilitation intervention in RC pathways. It was a single-center study and not sufficiently powered due to the natural history of the disease and attrition. It could be argued that the lack of power could introduce a bias, and we acknowledge this limitation. However, the differences found in physical function measured with leg muscle power were supported by the bioimpedance parameters and pointed towards improvements.

We do not have valid information on whether the intervention group continued exercising, and if so, to what extend. All patients were encouraged to follow the international recommendation of at least 150 min of activity of moderate tension per week after discharge. In addition, the intervention group was discharged with a plan for exercises based on the same exercise known from the pre- and postoperative interventions to further improve the convalescence.

We used surrogate markers for physical function (muscle leg power), nutritional status (handgrip strength), and body composition (bioimpedance); therefore, the results should be interpreted accordingly, although, all variables are recognized as highly valid markers in physical medicine [20,21,22].

The patients were enrolled 10 years prior to the study and before neoadjuvant chemotherapy was a recommendation. Naturally, the pathway has changed, including the introduction of robots in the surgical theater. However, this is the premise for all long-term follow-up studies. It could be argued whether the results remain relevant for today’s practice; we believe the results indicated a benefit for patients, and the outcomes demonstrated the role of prehabilitation in future RC pathways. However, high-powered RCT studies monitoring the patients more stringently are warranted to confirm validity.

## 6. Conclusions

The restoration of physical function is essential to obtain a full recovery, and prehabilitation in major bladder cancer surgery may improve physical function and support the maintenance of nutritional status and bioelectrical parameters one year after RC surgery. The results confirmed the increasing recognition of prehabilitation and its future role in the upcoming revision of the ERAS RC pathway with the potential to improve long-term outcomes. To further improve the efficacy of prehabilitation on patient outcomes and thereby improve the transition into the survivorship phase, the anabolic and metabolic interaction between physical exercises and the nutritional agent should be further explored.

## Figures and Tables

**Table 1:** Clinical and demographic covariates in 107 patients undergoing radical cystectomy at Aarhus University Hospital (DK) (2011–2014). To analyze and test for statistical differences between groups, the following test were used: the rank sum test (Wilcoxon) for categorical variables, the Pearson two-sided chi^2^ test for proportions, and Student’s *t*-test for continuous variables.

	Intervention *n* = 50	Standard *n* = 57	*p*
Gender Men, *n* (%) Women, *n* (%)	39 (78) 11 (22)	40 (70) 17 (30)	0.38
Age Mean [95% CI] Range	69 [66; 72] 46–85	71 [68; 73] 47–91	0.48
Maximum tumor stage, *n* (%) T1 T2 T3 T4	10 (20) 29 (58) 10 (20) 1 (2)	14 (25) 25 (44) 14 (24) 4 (7)	0.62
pN-stage, *n* (%) N0 N-pos Nx	35 (70 13 (26) 2 (4)	47 (83) 10 (17) 0 (0)	0.21
Pain (VAS 1–10), *n* (%) 0 1–3 4–5 ≥6	36 (72) 6 (12) 5 (10) 3 (6)	45 (79) 8 (14) 4 (7) 0 (0)	0.22
Charlson Comorbidity Index Score, *n* (%) No 1–2 Low 3–4 High ≥5 Severe	1 (2) 16 (32) 23 (46) 10 (20)	0 (0) 14 (25) 31 (54) 12 (21)	0.82
Nutritional Risk Score (NRS-2002), *n* (%) ≥3 “ at risk” <3	14 (28) 36 (72)	9 (16) 48 (84)	0.26
ASA-Score, *n* (%) 1 2 3 Missing	4 (8) 37 (74) 8 (16) 1 (2)	11 (19) 36 (63) 9 (16) 1 (2)	0.37
Body Mass Index BMI, mean [95% CI]	26 [25; 27]	26 [25; 27]	0.77
Nutritional intake (preoperative) Energy (kJ), mean [95% CI] Protein (g), mean [95% CI]	8897 [8294; 9501] 87 [81; 93]	8818 [8111; 9986] 86 [82; 92]	0.85 0.76
Smoker, n(%) Never <5 years ≥5 years Present Missing	10 (20) 16 (32) 5 (10) 15 (30) 4 (8)	9 (16) 12 (21) 15 (26) 18 (32) 3 (5)	0.38
Marital Status, *n* (%) Living with a partner Living alone Missing	31 (62) 16 (32) 3 (6)	32 (56) 21 (37) 4 (7)	0.56

**Table 2:** Efficacy of prehabilitation measured with leg muscle power and handgrip strength. One-year follow-up data after radical cystectomy in 107 patients. Aarhus University Hospital (2011–2014) (intention-to-treat approach).

Time	N (I/S)	Leg Muscle Power	*p*	Handgrip	*p*
Baseline (−14 days)	50/57	Mean—Watt/kilogram		Mean—Newton	
Intervention/standard		154.7/153.9	0.9	381/372.3	0.6
Day before surgery (−1)	47/53				
Intervention/standard		180.1/159.1	0.001	382.8/406.8	0.04
Discharge	46/49				
Intervention/standard		138.5/150.5	0.01	357.2/367.1	0.3
First visit day (35)	46/52				
Intervention/standard		155.3/151.9	0.8	359.9/381.2	0.04
Second visit day (120)	43/49				
Intervention/standard		180.8/160.7	0.01	398.3/366.1	0.1
Third visit day (365)	35/37				
Intervention/standard		176.3/156.5	0.04	424.3/392.1	0.1

**Table 3:** Efficacy of prehabilitation after radical cystectomy pathways. One-year follow-up on bioimpedance parameters expressed by mean differences between treatment groups using Student’s *t*-test in 107 patients undergoing radical cystectomy at Aarhus University Hospital (2011–2014) (intention-to-treat approach). * Statistically significant.

	Diff BMI [95% CI]	Diff Fat Mass of Total Body Weight, % [95% CI]	Diff Fat Mass, kg [95% CI]	Diff Lean Body Mass, kg [95% CI]	Diff Body Cell Mass, kg [95% CI]
**Baseline (−14 days)**					
Intervention vs. Standard	0.5 [−1.2; 2,2] *p* = 0.5	1.1 [−4.0;1.7] *p* = 0.4	−0.01 [−3.0; 3.0] *p* = 0.9	3.9 [ −1.0; 8.8] *p* = 0.1	1.4 [−1.9; 4.8] *p* = 0.4
**Day before surgery (−1)**					
Intervention vs. Standard	0.5 [−1.2;2.2] *p* = 0.6	−1.5 [−4.4; 1.4] *p* = 0.3	−0.9 [−4.0; 2.1] *p* = 0.5	2.3 [−2.6; 7.3] *p* = 0.3	1.5 [−1.8; 4.9] *p* = 0.3
**Discharge**					
Intervention vs. Standard	−0.1 [−1.9; 1.6] *p*= 0.8	−3.2 [−6.5; −0.02] *p* = 0.04 *	1.9 [−6.5; 10.4] *p* = 0.6	4.2 [−0.8; 9.4] *p* = 0.1	3.0 [−0.4; 6.5] *p* = 0.08
**First visit day (35)**					
Intervention vs. Standard	0.2 [−1.9;1.6] *p* = 0.8	−2.5 [−5.8; −0.7] *p* = 0.01 *	−0.8 [−3.9; 2.3] *p* = 0.6	10.5 [2.4; 23.4] *p* = 0.01 *	2.4 [−0.8; 5.7] *p* = 0.14
**Second visit day (120)**					
Intervention vs. Standard	0.1 [−1.8; 1.8] *p* = 0.9	−1.6 [−4.8;1.5] *p* = 0.3	−1.7 [−5.2; 1.9] *p* = 0.3	2.2 [−2.8; 7.3] *p* = 0.3	1.5 [−1.7; 4.9] *p* = 0.3
**Third visit day (365)**					
Intervention vs. Standard	0.5 [−2.1; 3.3] *p* = 0.6	−2.9 [−7.3; 1.3] *p* = 0.1	−1.6 [−6.6; 3.5] *p* = 0.53	6.7 [0.7; 14.35] *p* = 0.047 *	5.1 [0.3; 9.9] *p* = 0.03 *

## Data Availability

All data were collected by the bladder research group, anonymized, and entered into to an external database under the Central Region of Denmark.
